# Dravet syndrome

**DOI:** 10.1186/1824-7288-35-27

**Published:** 2009-09-08

**Authors:** Gemma Incorpora

**Affiliations:** 1 Pediatric Unit, Hospital " Civile - Paternò Arezzo", Ragusa, Italy

## Abstract

"Dravet syndrome" (DS) previously named severe myoclonic epilepsy of infancy (SMEI), or epilepsy with polymorphic seizures, is a rare disorder characterized by an early, severe, generalized, epileptic encephalopathy.

DS is characterized by febrile and afebrile seizures beginning in the 1st year of life followed by different types of seizures (either focal or generalized), which are typically resistant to antiepileptic drugs. A developmental delay from the 2nd to 3rd year of life becomes evident, together with motor disturbances and personality disorders.

Beside the classic syndrome, there are milder cases which have been called severe myoclonic epilepsy borderline (SMEB).

DS is caused by a mutation in the neuronal sodium channel gene, SCN1A , that is also mutated in generalized epilepsy with FS+ (GEFS+).

## Introduction

Dravet Syndrome (DS) was named after Charlotte Dravet who described this condition for the first time in 1978 [1] as severe myoclonic epilepsy, (SME) in a group of intractable epilepsy.

In the classification of ILAE [2] severe myoclonic epilepsy of infancy (SMEI) is included among " epilepsies and syndromes undetermined as generalized or focal".

Subsequent publications have reported patients with SME in families with GEFS+ and also the high familial occurrence for febrile and afebrile seizures, myoclonic astatic epilepsy and Lennox-Gastaut syndrome [3,4].

In 2001, Claes et al. [5] considering the presence of febrile seizures in patients with DS, examined the SCN1A gene, finding the novo mutations in probands, thus demonstrating that SCN1A is the main gene responsible for SMEI.

Other studies have confirmed the mutation of this gene in most, but not all patients affected. The same mutation has also been observed in concordant monozygotic twins [6].

In the last classification of Engel [7] SME is called D S and is included within the epileptic syndrome.

## Epidemiology

DS is a rare disorder.

The incidence is 1/40000 [8] o 1/20000 or 30000 [9] in the general population. Males are more frequently affected than females (2:1). Among patients with epilepsy, the incidence is 3%-5% in the first year of life and 7% by the age of 3 years [10].

A family history of epilepsy or febrile seizures is present in approximately 25% of cases.

A mutated SCN1A gene cannot be identified in approximately 20% of the patients who fulfil all the diagnostic criteria of the syndrome. Therefore it is possible that genes other than SCN1A such as GABAA-receptor gamma 2 subunit might be involved [11].

## Clinical findings and progression

Symptoms begin in the first year of life, or more often in the first six months, with tonic-clonic seizures almost always triggered by fever in an otherwise normal child. For this reason, the onset of the syndrome may be indistinguishable from febrile seizures.

A significant number of DS patients has a family history of seizures disorders or febrile seizures with phenotypes consistent with the GEFS+ spectrum, but the first febrile seizures episode is usually protracted. Between the 1st and 4th year of life additional febrile and afebrile seizures appear. The afebrile seizures are more frequently unilateral, tonic clonic, or secondary generalized, evolving later on towards other types of epilepsy including myoclonic, atypical absence, and atonic seizures, alternating partial seizures and convulsive status epilepticus. Recurrent status epilepticus which is always provoked by fever is a frequent event. Myoclonic seizures are not invariably present at onset. In the majority of cases, convulsive episodes have a focal origin. Generalized seizures are atypical absence with 2-3,5 Hz spike and waves discharges. Tonic seizures are very rare. Seizures, and in particular myoclonic seizures, occur very frequently during the first 4-5 years of life and are extremely drug resistant [12]. Some drugs such carbamazepine, lamotrigine, phenobarbital and phenitoin can exacerbate epilepsy.

Epilepsy has a tendency to improve over time.

Early psychomotor development is normal before convulsions starts, but developmental stagnation occur in the second year of life. Later a regression becomes evident, accompanied by hyperactivity, language deterioration and mental retardation and subsequently by pyramidal signs.

Some patients with DS show a " milder type" with less severe myoclonic epilepsy, less frequent tonic clonic seizures or status epilepticus, milder mental retardation and only transient ataxia (severe myoclonic epilepsy borderline SMEB).

DS is still a clinical diagnosis and the absence of a mutation in the SCN1A gene in symptomatic patients does not exclude the diagnosis.

Clinically, DS must be suspected in each otherwise normal child, with long lasting FS starting in the first years of life, followed by different type of afebrile seizures and neurodevelopment impairment. In these cases mutations in the SCN1A gene must be sought.

Today DS, the less severe borderline forms (SMEB) and GEFS+, can be considered as a continuum of the same condition [13,14].

The cognitive development of children with DS is constantly compromised. In the 1st year of life, patients are completely normal but in the 2nd year a slowing or a stagnation in psychomotor development is evident. In this period, the presence of behavioural disorders manifesting more often with hyperactivity and less frequent with autistic traits are also noted. Severe mental retardation is evident around the 5th year in almost all children, affecting all functional area. Sometimes in coincidence with a decrease in frequency of the epileptic seizures, the cognitive ability improves slghtly. In the first years of the disease, the motor and visuomotor functions are better preserved than language skills that are constantly compromised.

The aetiology of the cognitive and behavioural disturbances is not clear.

Risk factor for the presence of mental retardation are the onset of the epilepsy in the 1st year of life, high recurrence of seizures, status epilepticus and the presence of additional minor seizures.

Other neurological symptoms, such as progressive ataxia and pyramidal signs, give these patients a characteristic aspect.

## EEG findings

At the beginning of the symptoms the EEG is usually normal. With the recurrence of febrile and afebrile seizures, abnormalities become evident consisting of generalized, focal or multifocal anomalies, such as multifocal spikes, spike and waves, polyspike and waves discharges and a slowing of background activity. The intermittent light stimulation can be positive even before 1 year. Photosensitivity is not a constant feature during the course of the disease but in SMEI patients there is a photosensitivity spectrum because this response can persist and in some patients can be used as self-stimulation [15].

In general paroxysmal activity tends to disappear while awake but is prominent during sleep.

Ictal EEG anomalies, are suggestive of the syndrome and have been reported by Dravet e coll. [16] with video - polygraphic EEG recordings. The EEG discharge is bilateral but according to three modalities: 1) bilateral abnormalities from onset as slow spike or slow waves followed by a brief attenuation, 2) initially bilateral abnormalities becoming asymmetric, 3) bilateral and symmetric at their onset. The postictal EEG shows diffuse flattening or slowing.

Moreover, the peculiar clinical seizures and EEG features still not permit the real nature of this syndrome if multifocal or generalized.

## Brain imaging

Neuroimaging studies (CT and MRI), do not show any typical brain lesions even if brain abnormalities may exist [17]

In a recent review by Striano and coll. [18] on 58 brain MRIs in patients with SME, 22,4% of the patients showed abnormal findings consistent with cortical brain atrophy, cerebellar atrophy, hippocampal sclerosis, focal cortical dysplasia and ventricular enlargement. The authors concluded that the observed abnormal MRIs occurred more frequently in 39,15% of the patients without SCN1A mutation compared to 11,4% of those carrying a SCN1A mutation. These findings, if confirmed do not support the association in between prolonged febrile seizures and hippocampal sclerosis as previously reported by Siegler [19] in a retrospective study in 10 of 14 patients.

## Diagnosis

DS is still a clinical diagnosis that is mainly based on seizure history, clinical aspects, neurologic examination, EEG pattern and a long observation. Subsequently, DNA analysis with the evaluation of the SCN1A gene can confirm the diagnosis in the majority of cases.

In Appendix 1 are reported some diagnostic criteria. Currently, the diagnosis is suspected and performed earlier than before, because of the possibilities offered by molecular diagnosis.

## Relationship between DS and GEFS+

DS shares the same gene with GEFS+, wich was described by Scheffer and Berkovic in 1997 [3] in a large Australian family with epilepsy. It is an autosomal-dominant disorder in which a peculiar phenotype of febrile seizures (called FS+) has been demonstrated. These seizures start early and persist beyond 6 years of age. In the affected family coexist members with FS, FS+ and various types of afebrile seizures, such as GTCS, absence, atonic, myoclonic and also partial seizures. The afebrile seizures usually begin in childhood a continuum with febrile seizures being characteristic, occurring often after a variable seizure-free period. The prognosis is relatively benign without severe neurologic impairment. Affected members can exhibit more severe epileptic syndromes such as myoclonic astatic epilepsy (MAE), L G, and rarely DS.

GEFS+ is a heterogeneous genetic syndrome caused by more genes [3,11,13]. The first gene is SCN1B located on 19q 13.1 encoding the beta 1 subunit of the neuronal voltage- gated sodium channel. A second gene map in the region 2q21-q33 on SCN1A as in DS and finally a third SCN2A gene is located on 2q21-q23 encoding the alfa 2 subunit of the voltage-gated sodium channel.

Baulac et al [20] localized another gene of the gamma aminobutyric acid receptor (GABA A) on chromosome 5q34 but only few patients show this abnormality [21]

## Differential diagnosis

The differential diagnosis must consider simple febrile seizures, benign myoclonic epilepsy, LGS, MAE and the progressive myoclonic epilepsy.

Simple febrile seizures onset is mainly between 6 and 18 months, with tonic-clonic fits lasting < 15 minutes. The onset of FS in DS is within the first 6 months, they are more often unilateral, clonic and long lasting as febrile status.

Benign myoclonic epilepsy is accompanied by generalized spike and waves in the EEG and the myoclonic attacks are the only ictal manifestation. The neurological status is normal.

LGS starts later without FS with tonic attacks which are not evident in DS. The EEG shows diffuse 2/3 Hz spikes and waves. The differentiation between DS and MAE is very difficult, but in DS the drop attacks are not recurrent.

Progressive myoclonic epilepsies can be excluded because of the progressive worsening of symptoms and of the presence of metabolic dysfunction signs and progressive encephalopathy.

A skin biopsy in order to exclude ceroidolipofuscinosis (NCL) or a mitochondrial disease is often performed in patients with DS.

## Genetics

DS is a channellopaty caused by a mutation of the SCN1A gene with an average prevalence of 80% and ranging from 40% to 100% of the patients [22]. This difference is not explained by the ethnic origin, because it is present in Europe, Japan, Australia and South America, but perhaps is due to a lack of strict diagnostic criteria in the selection of patients. In Japan, the rate of mutation in SMEI patients is 77%-82%, in French, Italian, Australian and Canadian populations the incidence is much lower and only 35% of the cases are mutated.

SCN1A is found mutated in about 5% to 10% of GEFS+ patients.

The same mutation has also been found in SMEB and in single patients with severe myoclonic epilepsy and infantile spasms (IS) [23]. The SCN1A gene

Is located within a cluster of three sodium channel genes including SCN2A and SCN3A on chromosome 2q24. The beta subunit, SCN1B, was the first molecular link with GEFS+ [24]. Missense mutation in the alfa subunit, SCN2A, are associated with (BFNS) benign familial neonatal infantile seizures [25].

The SCN1A gene has a dominating role in the pathogenesis of DS with over 100 different mutations. The majority of these mutations are de novo (88%) but several are recurrent [26]. These mutations are spread throughout the entire protein with some clusters in the C-terminal and N-terminal ends and in segment 5-6 of the first 3 domains. SCN1A is the most clinically relevant epilepsy gene.

A few cases of DS children show a SCN1A microdeletion associated with mild dysmorphic traits and increased seizure resistance. This pattern has been called Dravet syndrome plus [27,28].

Other genetic or environmental modifiers or mosaics in parents as in other genetic diseases can be considered.

In Table 1 are reported the genes for DS and GEFS+.

**Table 1:** Sodium channel Gene for DS and GEFS+

**Gene**	**Epilepsy syndrome**
SCN1A	GEFS+ **SMEI (Dravet syndrome)**

SCN2A	GEFS+ BFNIS (benign familial neonatal infantile seizures)

SCN2B	GEFS+

## Prognosis

The prognosis is unfavourable in the majority of cases. All patients have drug resistant seizures that tend to persist throughout life. FS stop and the other seizures became less evident in the 2nd decade of life. The EEG anomalies are persistent.

Myoclonic seizures are very resistant to therapy and the period of the high recurrence of these seizures is associated with more severe neuropsychological impairment. Mental retardation is constant together with other neurological symptoms, like ataxia and spasticity. The convulsions are long lasting, status epileptics and the high recurrence of the episodes, particularly in the first years of life, deteriorate the brain function contributing to the neurological problems of these patients.

## Treatment

It is not know why this condition is so strongly drug resistant.

Some drugs such as phenobarbital, phenytoin, carbamazepina and lamotrigina may exacerbate seizures.

Valproate, clobazam and topiramate, are the drugs most frequently used with some result [12,14]. Stiripentol (not approved in US) is considered to be useful. Alternative therapies such the ketogenic diet, have been tried, but the results are controversial [29].

## Personal observations

In the last 6 years, we have evaluated 6 patients with a clinical picture compatible with the diagnosis of DS. Symptoms were severe in 5 patients consisting of severe hypotonia, dysmorphic features, and seizures since birth; a deletion of the entire SCN1A gene was found in 1 case with secondary generalization in another. Mutations in exons 20,21 and 23 of the SCN1A gene were found in 4 patients.

In our experience the two patients with deletions of the gene had clinically more severe epilepsy and both had dysmorphic features, severe microcephaly and marked psychomotor retardation.

Hyperactivity and attention deficit disorder, autistic traits, language disturbances and mental retardation were constantly present in all patients and persisted until adolescence.

## Conclusion

DS is an early infantile encephalopathy in which it is now possible to document the genetic background in a substantial number of cases.

Since the same genetic background has been demonstrated in other epileptic syndromes, it is possible that this condition is part of an epileptic continuum

## Competing interests

The author declares that they have no competing interests.

## Appendix 1

### Diagnosis of DS

Multiple seizures, frequently provoked by fever before 1 year

Unilateral or generalized clonic or tonic clonic seizures

Resistance to therapy

Normal development before seizure onset

Development delay at the age of 2 years

Normal interictal EEG in the early stages

Normal neurologic examination

Normal metabolic work up

Status epilepticus

Myoclonic seizures

Afebrile partial or secondary generalized seizures

Seizures provoked by fever after the age of 5
